# A new horizon in hydrovoltaics

**DOI:** 10.1093/nsr/nwaf371

**Published:** 2025-10-11

**Authors:** Jun Yin, Wanlin Guo

**Affiliations:** State Key Laboratory of Mechanics and Control of Mechanical Structures, Key Laboratory for Intelligent Nano Materials and Devices of the Ministry of Education, Institute for Frontier Science, Nanjing University of Aeronautics and Astronautics, China; State Key Laboratory of Mechanics and Control of Mechanical Structures, Key Laboratory for Intelligent Nano Materials and Devices of the Ministry of Education, Institute for Frontier Science, Nanjing University of Aeronautics and Astronautics, China

The global energy crises present the most critical challenges of our time, demanding innovative solutions. The Earth's natural water cycle, a fundamental process involving evaporation, transpiration, condensation, precipitation, runoff etc., holds immense and largely untapped potential for sustainable energy production. Hydrovoltaic technology has emerged as a transformative field, dedicated to directly converting the energy carried by water into electricity through interactions with functional materials. The simple architectures of hydrovoltaic devices make them exceptionally versatile and adaptable across diverse environmental conditions.

The past decade has seen the rapid expansion and maturation of hydrovoltaic science. Initial studies realized converting kinetic energy of water into electricity, leveraging the moving boundaries of an electrical double layer on graphene [1,2]. A key milestone was reached in 2017 when stable power generation from natural water evaporation was demonstrated for the first time [3]. These laid the groundwork for the ‘hydrovoltaic effect’ proposed in 2018 [4], giving birth to ‘Hydrovoltaics: new ways of harvesting electricity from water’ [5]. Since then, the field of hydrovoltaics has seen a remarkable surge in research and publications, marking a ‘new era’ of inquiry and innovation. This Special Topic of *National Science Review* assembles a collection of work that not only showcases significant technological and material advancements but also critically evaluates the field's foundational principles and future directions.

This Special Topic includes three perspectives, one research article and two reviews. In the perspectives, Wang *et al.* present a holistic vision for the ‘energy–water–air nexus’, offering a promising approach that integrates atmospheric water-harvesting technology with hydrovoltaic technology to create a combined system for sustainable water–electricity cogeneration [6]. Qu *et al.* present a critical analysis of core challenges in moisture-enabled electricity generation, including low efficiency and the trade-off between power density and generation duration, and calls for a shift in focus to enhancing interfacial ion–electron conversion efficiency [7]. Yao *et al.* call for a focus on sustainable mechanisms in moisture-based hydrovoltaic devices and warn against the development of ‘moisture-mediated chemical batteries’ that rely on irreversible redox reactions [8].

In the research article, Deng *et al.* present an engineering innovation that solves the long-standing challenges of cost and weight remaining for large-scale applications of droplet electricity generators [9]. They demonstrated a novel ‘nature-integrated’ device that uses water as both the bottom electrode and substrate, proving the concept's viability for land-free, large-scale applications.

In the reviews, Zhou *et al.* define and delineate the nascent field of bio-hydrovoltaics, tracing its evolution from non-living to living systems and highlighting the unique advantages of living systems like self-healing and dynamic adaptability [10]. Li *et al.* provide a comprehensive review of the fundamental science of interfacial water and its distinct properties, underscoring the critical need for a deeper, molecular-level understanding to enable future technological breakthroughs in hydrovoltaics [11].

Looking forward, as the field continues to evolve, we envisage hydrovoltaic science and technology not only as a source of sustainable and decentralized energy but also as a platform for future innovations, including hydrovoltaic ecology and hydrovoltaic intelligence. Hydrovoltaic ecology aims to create self-sustaining energy ecosystems. For instance, hydrovoltaic evaporation can generate power, provide cooling, and produce pure water simultaneously, paving the way for a new model of green and sustainable development. Hydrovoltaic intelligence refers to the development of novel brain-inspired intelligent devices based on hydrovoltaic science. A deeper understanding of the hydrovoltaic effect in biological systems like the human brain will help reveal the mysteries of human intelligence, opening innovative frontiers for artificial intelligence and brain-like technology. We are excited to see researchers from multiple disciplines push the boundaries and unlock the full potential of this fascinating field.
